# The predictive embodied mind: a case-based encounter with film aesthetics

**DOI:** 10.3389/fnins.2025.1583107

**Published:** 2025-06-30

**Authors:** Maarten Coëgnarts

**Affiliations:** ^1^Visual and Digital Cultures Research Center, Department of Communication Studies, University of Antwerp, Antwerp, Belgium; ^2^Research Unit Audiovisual Concepts, Luca School of Arts, Brussels, Belgium; ^3^Art History and Image Studies, Faculty of the Humanities, University of the Free State, Bloemfontein, South Africa

**Keywords:** embodied cognition, film style, image schemas, inferential processing, meaning-making, predictive processing (PP), visual aesthetics

## Abstract

In recent decades, the scientific study of the mind has experienced two significant conceptual shifts, each reshaping its research focus. The first, embodied cognition, questioned the objectivist framework of first-generation cognitive science by emphasizing that mental processes are deeply grounded in sensorimotor interactions with the world. Image-schemas have been suggested as one of the foundational conceptual elements underpinning this embodied grounding. The second shift is the revival of perception as an inferential process within the Predictive Processing (PP) framework. PP conceptualizes the mind as a predictive machine that minimizes error by actively anticipating sensory input through probabilistic computations. Rooted in Helmholtz’s theory of unconscious inference and updated as the Bayesian brain model, PP has been extended to diverse cognitive phenomena, including perception, emotion, and aesthetics. While its application to the visual arts and cinema has been relatively limited, this article seeks to advance this encounter by bridging PP with cinematic aesthetics. The article is structured into three sections. The first introduces the PP framework, outlining its core theoretical principles and linking it to the concept of image schemas. The second section reviews existing accounts of predictive processing in visual aesthetics, particularly in the non-temporal arts, and introduces a new case study of narrative painting that builds on Ladislav Kesner’s work on PP in art interpretation. In the final section we move to the temporal art form of narrative cinema, proposing that films engage the brain’s inferential processes by activating image schemas through deliberate formal design. By structuring sensory input in alignment with the brain’s predictive logic, cinematic works of art enhance both comprehension and the aesthetic pleasure derived from resolving predictions into coherent patterns.

## 1 Introduction

Over the years, the scientific study of the mind has undergone two significant conceptual shifts, each shaping its own research agenda: first, the realization that our mental life is deeply influenced by our physical interactions with the world; and second, the revival of the classical perspective of perception as an inferential process, highlighting the mind’s active role in interpreting sensory input. The first movement, known as embodied cognition, emerged as a direct response to the perspective held by first-generation cognitive science (Lakoff and Johnson, 1999, pp. 75–77). Following the cognitive revolution of the mid-twentieth century, this view posited that cognition is fundamentally non-perceptual, with the mental representations involved having no direct connection to the perceptual states that gave rise to them (e.g., Chomsky, 1957, 1965, 1975; Fodor, 1975). From this perspective, concepts are seen as symbols that derive their meaning, similar to language, by referring to a reality independent of the mind. This position, sometimes referred to as an “objectivist” paradigm (Johnson, 1987, 2007) assumes that meaning is derived from abstract representations that are largely independent of embodied experience. However, not all representational theories reject perception-based meaning-making; some incorporate indirect perception (e.g., Jacob and Jeannerod, 2003), while others—like models of extended cognition—acknowledge both representational and embodied processes (e.g., Clark, 2016). Embodied cognitivists challenge the objectivist paradigm by emphasizing that mental processes are not separate from our sensorimotor interactions with the world but are deeply grounded in them (e.g., Barsalou, 1999; Claxton, 2015; Gibbs, 2005; Johnson, 2007; Johnson and Schulkin, 2023; Lakoff and Johnson, 1980, 1999; Mandler, 2004; Shapiro, 2014; Tversky, 2019). A key concept that was introduced by George Lakoff and Mark Johnson in their 1987 books to couch the embodied foundation of thought and meaning was the notion of an image schema (Johnson, 1987; Lakoff, 1987; Lakoff and Johnson, 1999; see also Gibbs and Colston, 1995; Hampe, 2005). In cognitive semantics, image schemas are typically described as “structures of perceiving and doing” (Johnson, 2005, p. 16), or even more broadly as “redescriptions of perceptual events” (Mandler and Pagán Cánovas, 2014, p. 526). They are believed to play a significant role in the construction of meaning by linking patterns of the concrete realm of sensorimotor interactions to the abstract domain of higher-order reasoning (Johnson, 2005; Lakoff and Johnson, 1999). This idea is central to Conceptual Metaphor Theory (CMT) according to which abstract concepts, which are not directly tied to perceptual experience, are understood through mappings to concepts grounded in sensorimotor experiences (Lakoff and Johnson, 1980, 1999). For instance, spatial metaphors are frequently used to conceptualize abstract concepts like emotions and time (e.g., Boroditsky, 2000; Kövecses, 2000). While other schema theories—such as Bartlett’s schema theory (Bartlett, 1932) and Schank and Abelson’s script theory (Schank and Abeslon, 1977)—also operate at a higher level of abstraction than mental images, CMT offers a unique perspective by explicitly linking embodied patterns of thought with abstract reasoning. Furthermore, due to their pre-conceptual nature, image schemas are frequently depicted through diagrams that emphasize their key perceptual characteristics (Johnson, 1987; Lakoff and Johnson, 1999; Saslaw, 1996; Talmy, 1988). To represent these features more formally, scholars have recently developed the Diagrammatic Image Schema Language (DISL) (Hedblom et al., 2024). This system introduces a set of symbols to depict foundational conceptual primitives—the recurring elements of image schemas—and establishes principles for combining these components into complex image schemas and conceptual narratives. As such, DISL lends itself to potential spatial computational applications within the research program of embodied cognition.

While image schema theory furthers our understanding of how humans make sense of the world through structures of embodied knowledge, it does not provide a unified account that has the potential to explain our ability to impose structure on our sensorium. A growing perspective now prominent in cognitive science that seeks to address this issue is the Predictive Processing framework (henceforth, PP) (Hohwy, 2018, 2020). The transition from image schemas to PP can be motivated by recognizing that both frameworks emphasize structured pattern recognition in cognition. Whereas image schemas highlight recurring embodied patterns that structure perception and meaning-making, PP provides a functional account of how the brain actively predicts and processes these patterns in an inferential manner. This approach, the second significant conceptual movement, revives the old idea that the mind operates as a predictive machine—actively anticipating sensory input rather than passively registering it (Gregory, 1980; Helmholtz, 1860/1962). This perspective suggests that perceiving the richly structured and meaningful world around us requires the conscious ability to discern the most probable causes behind the ambiguous sensory inputs reaching our organs. Rooted in Helmholtz’s classical concept of unconscious inference, this perspective has been updated within contemporary neuroscience as the Bayesian brain model, which views the brain as a probabilistic system generating continuous predictions (Bar, 2009; Friston, 2009; Hohwy, 2013; Hohwy and Seth, 2020; Seth, 2021). At the same time, “there is vigorous debate,” as Hohwy (2020, pp. 10–11) points out, about how PP should be interpreted “in the context of embodied, enactive, extended, embedded cognition (4E cognition).” This debate results from the fact that cognitive science has traditionally favored mental representations above “direct perception.” Some scholars have emphasized that predictive coding remains deeply tied to our bodily interactions with the world, redefining key concepts from first-generation cognitive science—such as inference and computation—within an embodied framework (Clark, 2016, 2017; Michel, 2023; Newen, 2018; Seth, 2021; Venter, 2021, 2024). Andy Clark (2016), for example, has suggested that PP as a model supports his own framework of situated and extended cognition, where internal representations remain significant but are not overly elaborate. He proposes a pluralistic interpretation of PP, favoring efficient, simplified mechanisms over detailed, reconstructive representations. This approach emphasizes the role of Gibsonian affordances, with precision optimization serving as a key tool for interpreting and responding to environmental cues.

Leading proponents of PP have been portraying the predictive brain concept as a “grand unified theory of mind” capable of explaining a broad spectrum of mental and cognitive phenomena (Clark, 2013, p. 21; Friston, 2010). These include sensory and cognitive processes (such as perception and thought), motor functions (like action), mechanisms of attention and learning, and various emotional and psychological states (including emotion, affect, and overall well-being) (Frascaroli, 2024, p. 1). In the recent decade, this framework has also been extended to model aesthetics and symbolic forms across disciplines such as literature, music, and language (Frascaroli et al., 2024; Koelsch et al., 2019; Kukkonen, 2020; Menninghaus et al., 2024; Mortu, 2024; Vuust et al., 2022). This led some scholars to describe PP as “a vast and fast-growing research program that promises to deliver important insights into our aesthetic encounters as well as a wide range of psychological phenomena of general interest” (Frascaroli et al., 2024, p. 1). However, its application to the visual arts has been relatively modest by comparison. As Kesner (2024, p. 1) recently noted, this may reflect the fact that visual artworks lack “anticipatory structures and statistical regularities comparable to linguistic or musical syntax, which seem to provide a better fit with the hierarchical structure of predictive error minimization.” Yet, the role of prior knowledge in shaping perception has been a longstanding concern in art history. Gombrich (1960), informed by Bartlett’s schema theory, emphasized how viewers interpret artworks through learned schemas, highlighting the cognitive mechanisms that guide visual understanding (see also Ranta, 2021). While some significant progress has been made in applying PP to the non-temporal arts in visual aesthetics (Kandel, 2012; Kesner, 2014, 2024; Seth, 2019; Van de Cruys and Wagemans, 2011), its extension to the aesthetic experience of time-based art forms such as cinema is still in its early stages (e.g., Miller et al., 2024; Justus, 2025), notwithstanding some well-established and respected inferential accounts of perception and narrative comprehension in cognitive film theory (Bordwell, 1985, 1989; Hochberg, 2007; Hochberg and Brooks, 2007; Magliano et al., 1996). This article seeks to advance this encounter by offering a deeper exploration of film aesthetics and cinematic expressiveness within the PP framework while also emphasizing the crucial role that image schemas play in this process. The latter stems from the need, as also expressed by Kesner (2014, p. 10), to relate the concept of prediction (priors) to various other terms outside the PP framework that describe the diverse contents of mental representations. These representations play a role in recognizing and interpreting sensory input across the visual hierarchy, with image schemas being one such term. At the same time, PP could offer Conceptual Metaphor Theory a stronger neurological foundation. While theories by Feldman (2008) and Lakoff (2009, 2014) have investigated the neural organization of image-based conceptual metaphors, a challenge still remains in establishing a more precise cognitive computational basis for it (Michel, 2023, p. 39).

This article is organized into three main sections. First, we present the PP framework, briefly explaining its core theoretical principles and connecting it to the concept of image schemas. Next, we review existing accounts of inferential predictive processing in visual aesthetics, relating the framework to prior discussions of the non-temporal arts. Building on Kesner’s previous work linking PP to the interpretation of specific artworks (Kesner, 2014, 2024), we also discuss one new case-study which will further serve as a valuable basis for comparison when applying the predictive coding paradigm further to film aesthetics. This will be the focus of the third section, where we relate the cognitive neuroscience of predictive perception to the meaning-making processes underlying two specific case studies of cinematic art. In support of Frascaroli (2022, 2024), we propose that cinematic artworks are deliberately crafted to highlight the brain’s capacity to organize sensory input into meaningful patterns, thereby engaging its inferential processes. Within the formal design of cinematic art, image schemas are identified as central mechanisms driving this predictive inferential processing. By activating specific schemas through stylistic patterns of film form, cinematic works structure the sensory world in alignment with the brain’s predictive logic, enhancing both comprehension and the aesthetic pleasure that arises from resolving predictions into coherent configurations.

## 2 Predictive processing (PP) and image schemas

### 2.1 What is predictive processing (PP)?

**Figure 1:**
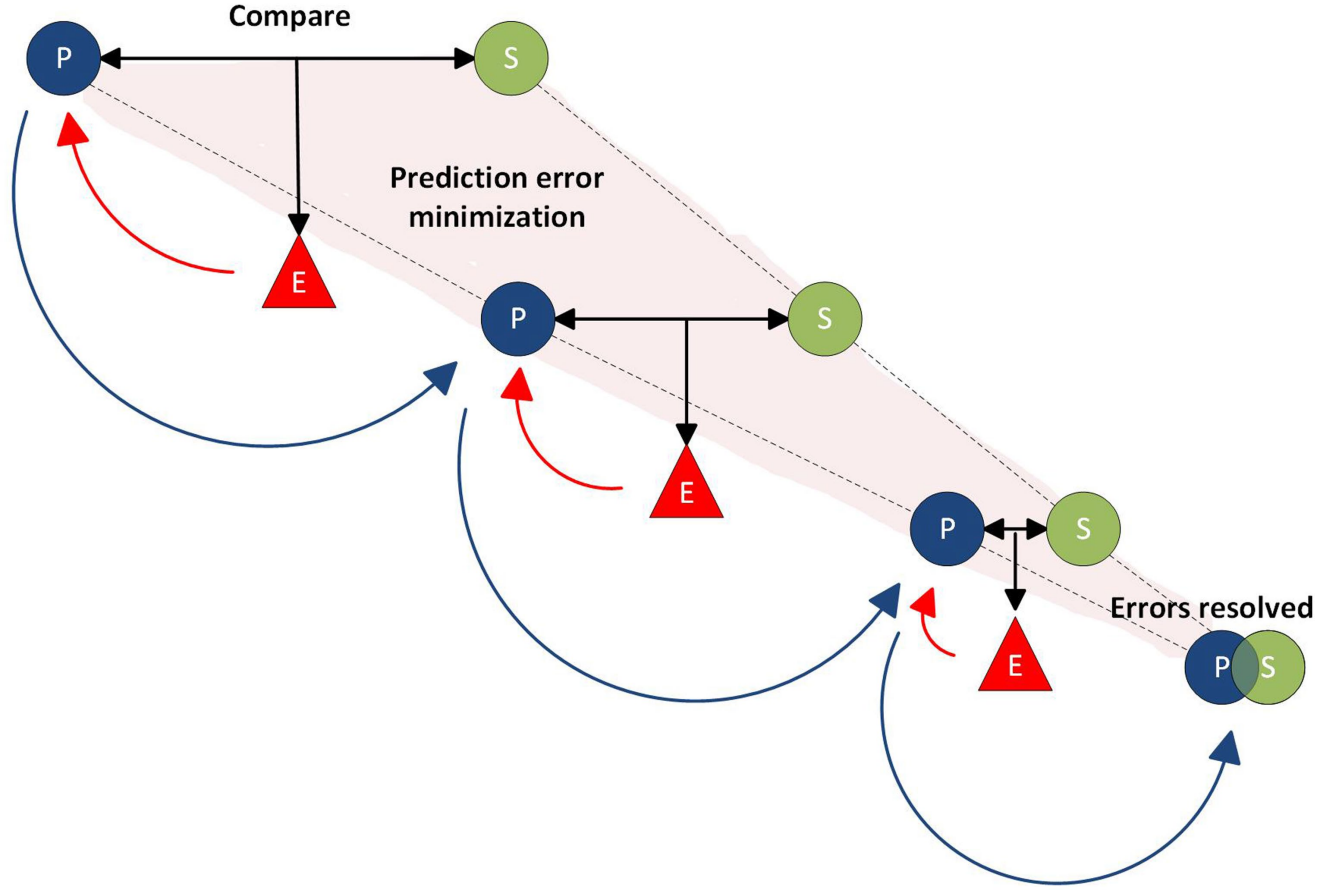
A schematic recreation of the mechanism of prediction error minimization as described by Seth (2021, p. 106). The brain generates top-down predictions about sensory input (P), which are then compared to the actual signals received by the sensory organs (S). Any mismatch between the predicted and actual signals produces prediction errors (E). These error signals, flowing in a bottom-up (outside-to-inside) direction (red vector lines), inform the brain to refine and update its predictions (blue vector lines). Ultimately, perception arises from the combined content of these top-down predictions, once the prediction errors have been minimized or “explained away” as effectively as possible.

Predictive processing (PP) provides a generative functional framework for understanding how the brain actively predicts and interprets sensory information. As such, it is a “theory about the *mechanisms* by which brains accomplish perception” (Seth, 2021, pp. 106–107). This differs, for example, from Anil Seth’s “controlled hallucination view,” which takes the theory of PP further to explain the nature of conscious experiences, what he refers to as “the *phenomenological properties* of conscious perception” (Seth, 2021, p. 107). A core principle of PP is that the brain operates as a predictive mechanism, structured along the hierarchical organization of cortical sensory areas. Each level generates hypotheses, so called “best guesses,” about the causes of the sensory signals that constitute our noisy and ambiguous world, and updates these predictions based on the error—or mismatch—between expected and actual data (Seth, 2021, p. 100). This iterative process, from higher-level models (e.g., the prediction to see a “gorilla” as in Seth’s example) to lower-level sensory signals (e.g., predictions about shapes, colors, and edges), allows the brain to construct a coherent and structured representation of the world (Frascaroli, 2024, p. 2; Seth, 2021, p. 104). According to Lisa Feldman Barrett, the functioning of predictive processing can be pictured through the structure of a “prediction loop” (Barrett, 2017, pp. 62–63). In this loop predictions act as simulations of sensations and movements, which are then compared to the actual sensory input from the environment. When the predictions align with the sensory input they are confirmed, and the simulation becomes your conscious experience, enabling us to engage with a structured world filled with objects, people, and an orderly flow of events. Perception thus occurs through an ongoing process of “prediction error minimization” (see Figure 1), where discrepancies between the brain’s predictions and sensory input (prediction errors) are reduced or “explained away” to the greatest extent possible (Seth, 2021, p. 106). If there is a mismatch, the brain must resolve the resulting errors. Through this ongoing process of prediction and correction, our brain continuously constructs and updates our mental model of the world (Barrett, 2017, p. 62).

The principles established by Reverend Thomas Bayes offer a powerful framework for modeling the structure of the predictive loop (Seth, 2021, p. 102). According to these principles, the brain constantly updates its predictions by integrating prior knowledge (or beliefs) with new sensory evidence. In this model, prior probabilities represent the brain’s expectations based on past experiences (so called priors), while sensory input provides the likelihood of those predictions being accurate. The brain then combines these two sources of information to form a posterior probability, which refines its model of the world. This process of updating predictions ensures that the brain continuously adjusts its mental model to better match the environment, minimizing prediction errors over time. As such Bayesian probability provides a mathematical framework for understanding how the brain handles uncertainty and adjusts to new information in real-time. In statistical terms Bayesian probability can be effectively represented using the Gaussian probability distribution, commonly referred to as the normal distribution (Seth, 2021, pp. 104–105). This statistical model is widely recognized for its bell-shaped curve, characterized by a central peak at the mean value, which represents the most likely outcome. The spread of the curve, determined by its standard deviation, reflects the degree of uncertainty or variability around the mean. In the context of Bayesian reasoning, the Gaussian distribution is particularly useful for modeling probabilistic inferences because it allows for the continuous updating of beliefs based on new evidence. By assigning higher probabilities to values closer to the mean and progressively lower probabilities as values deviate from it, the normal distribution aligns with the Bayesian principle of weighting prior knowledge against incoming data.

### 2.2 Predictive processing and image schemas

This brief picture of PP makes clear that the brain does not simply respond to sensory input; rather, it actively generates predictions based on prior experiences, which are shaped through embodied interactions with the environment. As early as 1934, American philosopher John Dewey articulated the significance of the latter by defining the organism-environment interaction as “the live creature,” highlighting the non-dualistic principle that conceptual organization and learning are fundamentally grounded in embodied experience. As he writes: “There are common patterns in various experiences, no matter how unlike they are to one another in the details of their subject matter. (…) The outline of the common pattern is set by the fact that every experience is the result of interaction between a live creature and some aspect of the world in which he lives” (Dewey, 1934/2005, p. 45). Similarly, in the 1970s, psychologist James J. Gibson introduced the concept of affordances, referring to the perceived possibilities for action that environments offer, provide or furnish, “either for good or ill,” based on the interaction between an organism’s abilities and its surroundings (Gibson, 1979/2015, p. 119). More recently, neuroscientist Anil Seth has echoed these classical views in his “Beast Machine Theory,” arguing that “we cannot understand the nature and origin of our conscious experiences, except in light of our nature as living creatures” (Seth, 2021, p. 174).

The connection between the organism and its environment raises the question of how knowledge gained from bodily interactions (priors) – Dewey’s “common patterns” – is represented in a way that facilitates predictive processing. Image schema theory offers one useful model for addressing this question, as it relates directly to the content of mental representations that underlie the PP framework (see also Kesner, 2014, p.10). First introduced by Lakoff (1987) and Johnson (1987) in the 1980s, the concept of image schema was developed to explain the bodily grounding of mental representations (see also Lakoff and Johnson, 1999). An image schema, Johnson (1987, p. 29) writes, “*is a recurrent pattern, shape, and regularity in, or of our ongoing ordering activities*.” They are “*structures for organizing* our experience and comprehension.” Building on this foundation, developmental psychologist Jean Mandler expanded the idea in the early 1990s with her theory of “perceptual meaning analysis” (Mandler, 1992, 2004, 2005). According to Mandler, perceptual information provided by the environment is conceptualized from an early age as infants extract “the spatial and movement structure into image-schematic form to represent them” (Mandler, 2005, p. 138). She describes image schemas as “dynamic analog representations of spatial relations and movements in space” (Mandler, 1992, p. 591). Underlying these schemas are so-called “spatial primitives,” “the first building blocks that allow us to understand what we perceive: path, container, thing, contact, etc.” (Mandler and Pagán Cánovas, 2014, p. 526). The conceptual primitive of a container (a bounded region in space), for example, has an inferential logic, based on the notions of an inside, an outside, and a boundary, that shapes our understanding of the world by providing a framework through which we make predictions, draw conclusions, and reason about events (Lakoff and Johnson, 1999, pp. 31–32).

**Figure 2:**
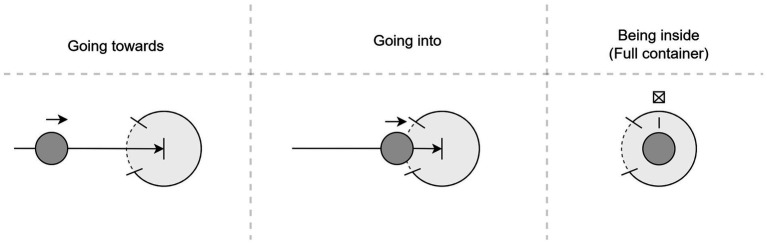
DISL example: full image schema scenario of object going_into a container (recreated after Hedblom et al., 2024, p. 22).

Despite their name, image schemas are neither images nor schemas of (mental) images but rather semantic constructions. According to Johnson (1987), image schemas operate on a higher level of abstraction than mental images. They consist of a small number of parts, which allows them to be used to “structure infinitely many perceptions, images, and events” (Johnson, 1987, p. 29). Nevertheless, image schemas are frequently explained and illustrated with the help of diagrams, which show the salient features of a given image schema (e.g., see examples in Coëgnarts, 2019, 2023a; Dewell, 2005; Hedblom, 2020; Hurtienne et al., 2015; Johnson, 1987; Saslaw, 1996; Talmy, 1988). More recently, scholars have developed a more formal visual language to capture the semantic entailments of image schemas visually (Hedblom et al., 2024). They call this language the Diagrammatic Image Schema Language (DISL). This language draws its inspiration from comic strip conventions to represent image schemas and their integrations. Similar to comics, DISL uses sequences of panels, termed “strips,” to depict narratives concisely with each strip comprising panels that illustrate specific image-schematic scenes. These scenes typically include objects, their states, and relationships, and the strip as a whole conveys a temporally extended narrative by sequentially presenting these evolving scenes. Figure 2, for example, shows the full image schema scenario of the event “going into a container” (Hedblom et al., 2024, p. 22).

**Figure 3:**
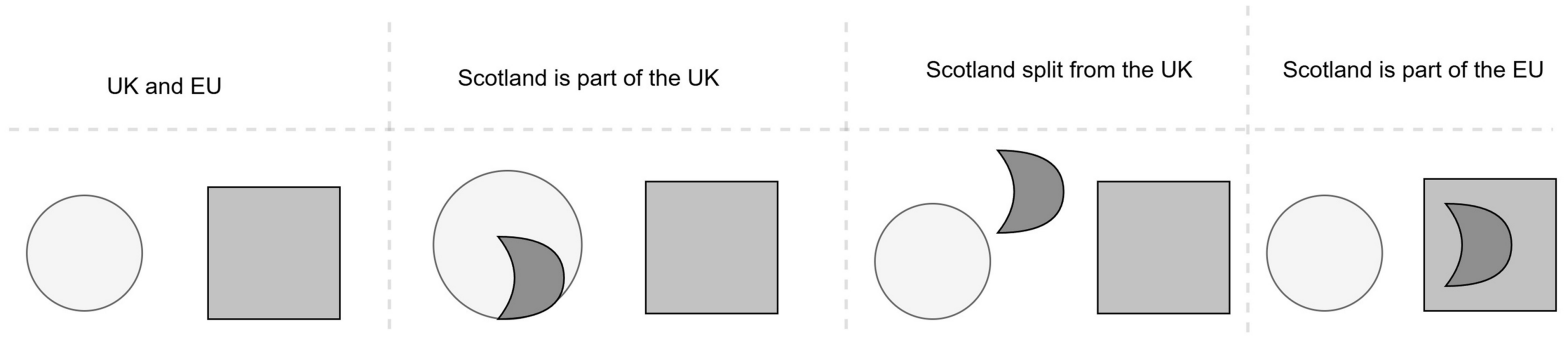
DISL provides a well-defined language for analyzing narratives such as “Scotland splits from the UK and joins the EU” (recreated after Hedblom et al., 2024, p. 24).

As the authors further argue, DISL can be extended in order to analyze and represent more complex abstract events or narratives. In their study, they demonstrate the expressivity of DISL by analyzing complex cases such as the verbal expression “Scotland leaves the UK and joins the EU” (Hedblom et al., 2024, p. 24). This narrative involves two objects, each comprising multiple parts: the United Kingdom and the European Union. Initially, Scotland is part_of the UK, but it splits from the UK and subsequently joins the EU. The underlying image schematic logic can be represented as in Figure 3.

From the perspective of PP, DISL’s approach aligns with the brain’s reliance on hierarchical predictions to process and understand complex narratives. The sequential representation of image schemas in DISL mirrors how the brain anticipates transitions between states or relationships based on prior knowledge and context. For instance, in the narrative of Scotland’s political realignment, PP could model the changing affiliations as a series of prediction error minimizations, where the mismatch between expected and observed states (e.g., Scotland’s part_of relationship with the UK) is resolved through updated predictions.

Integrating image schemas with PP requires further clarification regarding the cognitive levels at which these structures function. While image schemas are described as embodied conceptual structures that support abstract reasoning, they are generally considered to operate “beneath the level of conscious awareness,” at the level of what Johnson (2005, p. 22) calls the “Cognitive Unconscious”. Similarly, PP posits that predictions span a hierarchical range, from low-level sensory inferences to abstract conceptual reasoning.

This integration suggests that DISL’s schematic logic could serve as a valuable tool for modeling how abstract and dynamic narratives are processed within the PP framework. Further below, we will illustrate how this extends to two complex narratives found in film art. However, before doing so, let us first explore the connection between the PP framework and our experience of non-temporal visual artworks.

## 3 Linking predictive processing to the visual arts

From the point of view of the humanities, the PP framework prompts the question of how it can offer meaningful explanations for our experiences of aesthetics and symbolic forms. As Seth (2019, p. 398) asks: “What can be said about aesthetic experiences from the perspective of the predictive brain?” Since the PP framework was originally conceived as a general functional theory of brain function, a significant challenge remains how to connect this framework at the neural level to the cognitive-psychological level, where the subjective experience of aesthetics and symbolic forms resides (Kesner, 2014, p. 1). In an effort to address this challenge within the domain of visual aesthetics Van de Cruys and Wagemans (2011) proposed their “tentative prediction error account of visual art” (TPEA). With this model the authors attempted to leverage PP against the philosophical question of why aesthetic encounters are pleasurable and engaging (see also Frascaroli et al., 2024). Their model suggests that the brain’s prediction errors—discrepancies between expected and actual sensory input—are central to aesthetic experience. By delaying prediction confirmation, so the argument goes, artists create situations where the viewer encounters inconsistencies or surprises, which trigger arousal aimed at resolving these errors. This tension between expectation and sensory input fosters emotional engagement, as viewers work through these discrepancies, intensifying their emotional connection with the artwork. The mental effort required to resolve these prediction errors is key to experiencing perceptual pleasure, as it leads to a sense of coherence and satisfaction when the brain integrates sensory input into a meaningful whole (a Gestalt). In other words, aesthetic pleasure emerges from the positive emotional response we experience when we are more successful than usual in interpreting and understanding our environment. In this sense aesthetic pleasure can be understood as “the mark of a cognitive and existential conquest” (Frascaroli et al., 2024, p. 4). Ideally, the goal of perceiving is to go through a successful, satisfying process of understanding that ultimately leads, in a hierarchical fashion, to a satisfactory grasping of the meaning.

This process involves predictions at different levels of the visual processing hierarchy: from lower-level predictions involving object recognition to higher-level, semantic predictions (Kesner, 2014). At the lower stage, predictive processing begins with raw sensory data. The brain receives visual input from the environment (e.g., light patterns hitting the retina) and processes basic features like color, edges, motion, and shape. In the next stage, the brain starts grouping sensory features into recognizable objects or scenes. This “rush to the object,” as Kesner (2014, p. 3) calls it, includes recognizing familiar objects, faces, and patterns. The brain compares incoming sensory data with stored templates or prototypes. Moving a level up, higher-order meaning-making begins. The brain integrates the sensory data into a broader context, considering not just the identity of objects but their relevance and meaning in a particular situation. The brain anticipates the context or scenario, such as predicting the emotional response or the purpose of an object based on past knowledge and social or cultural context. The highest level encompasses abstract reasoning and the formation of complex, higher-order meanings, where, as embodied cognitivists have pointed out, the role of image schemas becomes particularly significant. As Johnson (2005, p. 15) notes: “As patterns of sensory-motor experience, image schemas play a crucial role in the emergence of meaning and in our ability to engage in abstract conceptualization and reasoning that is grounded in our bodily engagement with our environment.” As cognitive tools they help bridge the gap between the sensory, physical experience of visual art and its abstract, symbolic meanings, thus enabling viewers to make connections between the artwork and their own felt experiences. For example, when viewing a piece of visual art, an individual’s brain might predict the physical relationships between objects based on familiar image schemas (like containment or balance), adjusting their perception based on the artwork’s manipulation of these schemas. Art that plays with or distorts these schemas may disrupt the viewer’s predictions, eliciting surprise or deeper engagement, as the brain has to reconcile the discrepancy between its predictions and the visual input. Moreover, since image schemas serve as carriers of abstract meaning, as the case studies below will further illustrate, they serve as tools that facilitate the entire process of meaning-making.

As Kesner (2014, p. 2) observes in his discussion of the predictive model of visual arts, one of its key strengths is that it provides a much more robust explanatory framework compared to neuroscientific models of art experience that tend to focus solely on a bottom-up account of visual art works (Freedberg and Gallese, 2007; Shimamura, 2013; Zeki, 1999). Furthermore, because this model aligns with well-established and respected art historical theories, it encourages us to reinterpret these classical views through the explanatory lens of predictive coding. More recently, Seth (2019) has engaged with this challenge in relation to Gombrich’s concept of the beholder’s share and the viewer’s inferences in perception (see also Kandel, 2012, p. 205).

At the same time, however, we must be cautious not to overestimate the explanatory power of the PP framework. As Kesner (2014, p. 2) states, “even if predictive coding were to become a grand unified theory of brain function, there are compelling reasons to remain skeptical about its potential to evolve into a comprehensive biological theory of art and art perception.” One fruitful way to foster dialogue between PP and visual art theory is therefore by abandoning the commitment to a “Grand Theory” for what art philosopher and film theorist Noël Carrol calls “piecemeal theorizing,” an approach that aligns with what his colleague David Bordwell refers to as middle-level research (Bordwell and Carroll, 1996). Piecemeal theory refers to an approach to philosophical inquiry or theory-building that emphasizes gradual development and refinement through the study of specific, concrete cases, rather than attempting to construct a comprehensive or all-encompassing theory from the outset. In this approach, theorists build their understanding incrementally, addressing particular problems and observations as they arise, and refining their theories based on these findings. This is exactly the approach that Kesner (2014, 2024) has adopted in his efforts to refine the PP model through in-depth and insightful case studies of specific types of visual art objects, and this will also be the approach that we will pursue here.

In the following subsection, we will explore and demonstrate the application of the PP model to a neo-classical figurative (representational) painting: Jacques-Louis David’ *The Oath of the Horatii*. This case study can be seen as a new addition to those previously presented by Kesner (2014, 2024) whose own discussions provided a valuable template for analyzing David’s painting through the lens of predictive coding. The discussion of a narrative painting will provide a valuable and insightful frame as we move on to the next section, where we address the challenge of extending the explanatory framework of PP to the aesthetic experience of narrative cinematic art works.

### 3.1 Case-study: *the Oath of the Horatii* (David, 1784–85)

*The Oath of the Horatii*, a monumental painting measuring 13 by 10 feet, is widely regarded as Jacques-Louis David’s masterpiece and a cornerstone of the neoclassical movement. Created in 1784–85, it was showcased at the Paris Salon in 1785, on the eve of the French Revolution. It provides a compelling example of perceptual stability, where the painting’s clear representational nature enables the viewer to quickly identify the depicted objects. Moreover, unlike the figurative case discussed by Kesner (i.e., Vincent Desidero’s *Spiegel Im Spiegel*), where the representational status remains more opaque, further engagement with its compositional and perceptual features leads to a more profound understanding of its subject or theme. At the lower level of the prediction hierarchy, a motivated viewer—defined by Kesner (2014, p. 4) as someone “willing and able to endure more than a fleeting encounter with the painting”—can have their initial response understood through the mechanisms of sensory prediction that underlie basic recognition. The painting’s low spatial frequency information, which encodes its overall structure and composition, activates object and category recognition. This, in turn, provides a predictive template that directs further sensory processing, focusing on high spatial frequency details to reveal the finer elements of the work. The viewer encounters no difficulty in identifying the individual figures within the pictorial space—unlike the potential challenges posed by abstract avant-garde art. When prior knowledge is absent, as with modernist formal innovations that sometimes deviated from the familiar pictorial principles of the Renaissance, incomprehension arises (Kesner, 2024, p. 3). This explains why, at the turn of the twentieth century, audiences struggled to recognize forms in expressionist paintings—they lacked the preexisting frameworks needed to verify and interpret them. In Predictive Processing terms, “the sensory data were too noisy to be successfully minimized,” thus leading to “negatively valenced affect, such as confusion, anger, and boredom” (Kesner, 2024, p. 4).

**Figure 4:**
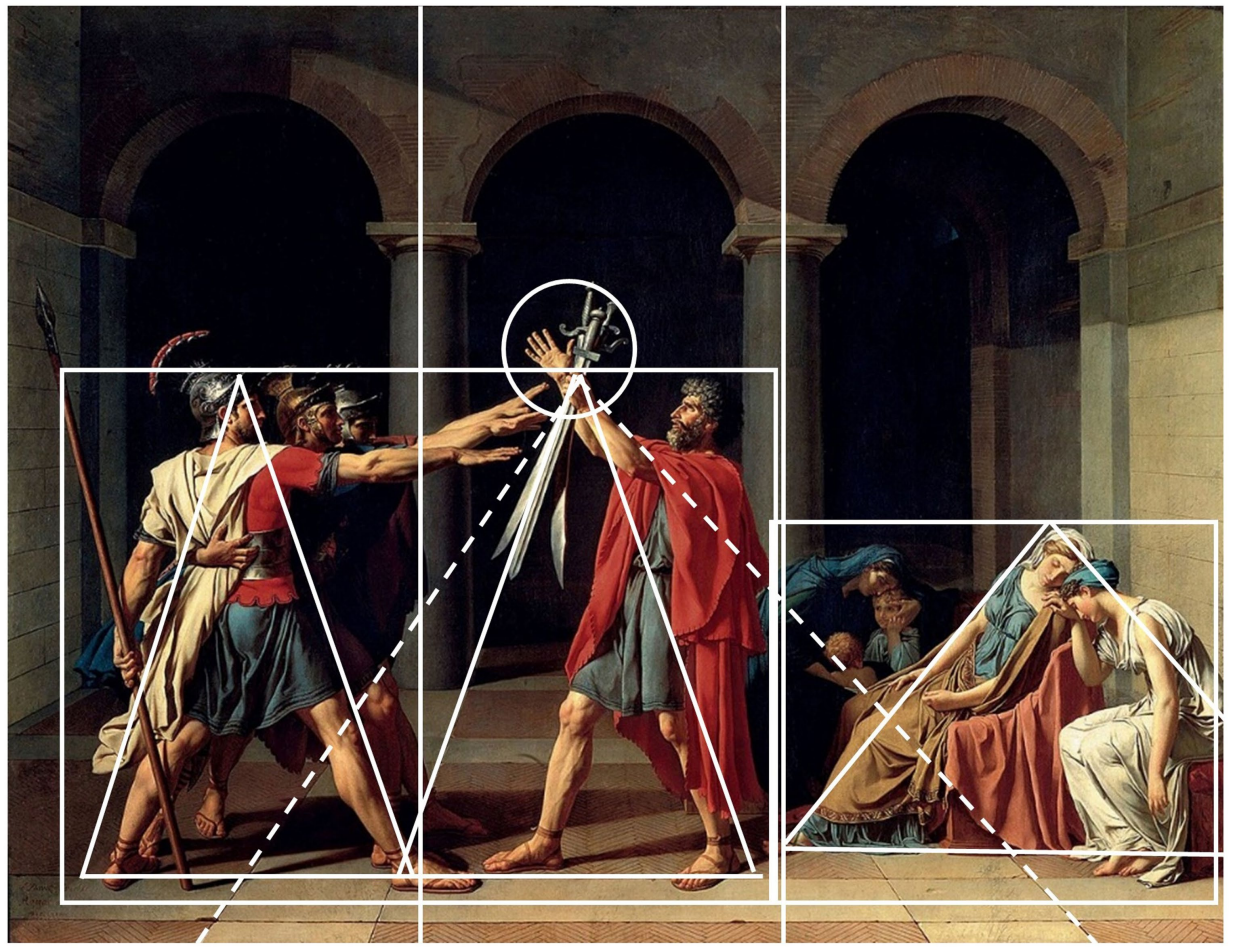
Structural analysis of Jacques-Louis David’s *The Oath of the Horatii* (1784, oil on canvas, courtesy of Louvre, Paris), highlighting its hidden compositional framework. The underlying geometric structure, by Arnheim described as the painting’s “hidden skeleton,” organizes the figures into distinct yet interconnected spatial zones, guiding the viewer’s perceptual and interpretative processes. The triangular configurations and container dynamics shape the scene’s narrative tension, reinforcing the contrast between the rigid, oath-taking men and the grieving women. By mapping these compositional cues, the diagram reveals how the painting’s formal design anticipates and structures the viewer’s higher-order semantic engagement.

This, however, is not the case here where the viewer can easily identify the objects. As graphically indicated in Figure 4, the composition clearly delineates its subjects: the three resolute brothers, unified in their stance, on the left; the father, prominently positioned in the center, holding three swords aloft; and the mourning women on the far right—Camille, dressed in white symbolizing purity, Sabine in gold and blue, accompanied by a nurse and two children. As Berg (2007) notes, while many in the French public were familiar with the story of Horace through Corneille’s popular play, even viewers unaware of it or the painting’s title could easily grasp key themes including allegiance to a higher ideal (raised arms), military service (swords), solidarity (converging arms of the brothers), male strength (muscular forms), female vulnerability (reclined women), generational hierarchy (older central figure), and patriarchal authority (father’s central position and raised hand as the focal point).

While most objects and themes in the painting are easily identifiable, unresolved questions remain—such as the relationships between the visual elements and the scene’s overall meaning. Prolonged engagement with the depicted scene initiates a predictive error minimization (PEM) cycle, prompting conscious reflections such as in this case: Why are the brothers swearing an oath? What is their relationship to the central figure? How do gender contrasts highlight societal roles? What does the father’s central position indicate about authority? How does composition enhance tension and meaning? As Kesner (2014) further explains through the PP framework, while the content of the pictorial scene at the level of individual objects is almost entirely resolved as observed information is iteratively reconciled across various levels of visual processing, reducing prediction error and leading to a single perceptual interpretation, identifying individual objects alone does not provide inferences about their relationships or the overall meaning of the scene. This initiates a series of “higher-level semantic predictions, which unfold through an ongoing exploration of the painting and concern above all the nature of the interaction between the depicted figures” (Kesner, 2014, p. 5).

What affords this higher-level exploration are the abstract perceptual patterns embedded in the art work and which we can further explain through Rudolf Arnheim’s theoretical framework of perceptual dynamics (Arnheim, 1988a; see also Winner, 2019, Chapter 5). His analysis of another neoclassical painting by the French painter Pierre-Narcisse Guérin offers an apt summary of his perspective, linking theme with the painting’s underlying structural skeleton: “An episode of human life is reduced to a configuration of visual forces, which outlines the conflict diagrammatically. However, this diagram is not presented directly or overtly. We perceive it through the painted figures of the actors, because perception is not simply the recording of shapes, but the understanding of the structure underlying the appearance of objects” (Arnheim, 1988a, p. 590).

What Arnheim refers to as visual forces are the “directed tensions’ in our acts of perception. They represent the “perceptual forces” inherent in shapes, configurations, colors, and movements in the visual world. As Arnheim states, these dynamic properties are so fundamental that we can say “*visual perception consists in the experiencing of visual forces*” (Arnheim, 1954/1974, p. 412). This applies to both natural objects (like the dynamic curve of an ocean wave) and works of art (such as a painting). However, the key difference is that natural objects were not intended to embody an abstract pattern or configuration of forces, while artworks such as David’s painting are specifically designed to be perceived and convey meaning. Arnheim notes that natural objects only “carry visual form impurely and approximately,” while artists aim to create “the strongest, purest, most precise embodiment of the meaning that, consciously, or unconsciously, he intends to convey (Arnheim, 1969, pp. 270–271). As Arnheim writes, “a timeless medium such as painting portrays human life as a closed system in which all relevant forces are shown together in configuration, each in its characteristic direction and appropriate strength” (Arnheim, 1988a, p. 590).

David’s painting exemplifies this interplay of perceptual form and ideological/thematic content through its composition and the spatial arrangement of the figures. Bryson (1981, p. 79) has noted that the perceptual form of the painting centers around a perceptual tension between the frieze-like arrangement of the figures and the converging perspective lines of the floor tiles, creating a sense of depth (see also Berg, 2007, p. 47). The overall effect on the viewer is a sense of balance, defined by Arnheim as “the dynamic state in which the forces constituting a visual configuration compensate for one another. The mutual neutralization of directed tensions produces an effect of immobility” (Arnheim, 1988b, p. 225). Moreover, the vanishing point, positioned at the intersection of the three swords in the father’s left hand, pushes “attention back toward the frontal plane, back toward the thematic action and interaction” (Berg, 2007, p. 47). The background, especially the arches, divides the composition into three distinct sections (“containers”)—left with the brothers, center with the father, and right with the women and children—while this tripartite division is further emphasized vertically by the heads of the seated women occupying a line below the heads of the standing man and the top of the painting. As Berg (2007, p. 48) has observed, this tripartite division affords the ideological content of the painting to the spectator as it conveys a value system strongly based on patriotism. This organizing principle is echoed in the shapes of the male figures, which geometrically represent the number three, reinforcing their alignment with the norm, while the flattened oval shape of the women and children sets them apart.

While in timeless media such as painting perceptual dynamics afford this higher-order semantic predictions, we will further elaborate below how image schemas serve a similar function in time-based media such as film. Both mechanisms afford a productive “hermeneutical cycle in which an encounter with a work of art” activates “a complex interplay of predictions that span every level within the hierarchical structure of the mind/brain, from sensory/recognitional to high-level semantic predictions” (Kesner, 2024, p. 3).

## 4 Predictive processing and film aesthetics

Moving to the realm of narrative cinema, we are confronted with an art form that represents “a series of events in time” (Levinson and Alperson, 2015, p. 162). This compels the viewer to adopt an entirely different viewing strategy (Higgins, 2011, p. 113). From the compositional focus of the aesthetic observer in painting we move towards “the practical, goal-oriented focus of the cinemagoer,” who navigates the unfolding narrative from “the constantly changing vantage point of the present moment” (Arnheim, 1988b, p. 234). The Event Indexing Model (henceforth, EIM), initially introduced by Zwaan and colleagues to explain how we comprehend written stories (e.g., Zwaan et al., 1995; Zwaan and Radvansky, 1998), and later extended to film narratives (e.g., Magliano et al., 2001; Magliano and Zacks, 2011), offers a dynamic inferential framework for understanding how readers and viewers process narratives across written and visual media. According to the cinematic application of this model, viewers track key dimensions of the story—time, space, characters, causal relationships, and goals—updating their mental maps as events unfold onscreen (Magliano et al., 2001). Predictive processing explains this process by suggesting that the brain continuously generates predictions about upcoming events based on prior knowledge and cues from the film. When these predictions are violated, prediction errors occur, prompting the brain to adjust its understanding of the narrative. For example, in a film with a complex plot, viewers predict the sequence of events, causal connections, and character actions. If an unexpected twist or shift in time or space occurs, the brain experiences a prediction error and updates its mental representation of the narrative, allowing viewers to make sense of the story. However, not all films actively distort or manipulate predictive structures. Some cinematic works, particularly within certain genres, rely on reinforcing well-established predictive patterns rather than subverting them. Genre expectations, for instance, play a crucial role in shaping viewer anticipation, as different film traditions condition audiences to predict narrative structures in specific ways. This means that prediction mechanisms are not only driven by low-level perceptual cues but also by high-level cultural and stylistic conventions (Bordwell, 2008).

EIM, as recently further elaborated by Loschky et al. (2020) into the Scene Perception and Event Comprehension Theory (SPECT) operates primarily at the level of understanding visual narratives. Viewers predict transitions between scenes, shifts in temporal structure, and changes in character intentions based on prior exposure to filmic conventions. The degree to which filmmakers manipulate these anticipatory processes—through editing, pacing, or cinematographic techniques—determines how predictive expectations are confirmed or violated. This means that a film’s artistic construction is key to understanding how predictive models function in a cinematic context.

However, while the EIM focuses on the perception and understanding of films, it does not fully address the more interpretive or metaphorical dimensions of cinematic meaning-making, at a level comparable to David’s painting as discussed above. For predictive processing to fully address this aspect, it must go beyond tracking basic events and move from processing literal events to the representation of more abstract ideas in art. This includes understanding how image schemas function in cinematic works not merely as cognitive structures but as artistic devices that can be actively shaped, distorted, or reinforced for aesthetic effect. Whereas perceptual dynamics play a significant role in facilitating this process in non-temporal art forms such as painting and sculpture, we will now illustrate, through two case-studies, how a structured unfolding of image schematic logic serves a similar purpose in cinematic art. This excercise follows previous efforts to link image schema theory to the interpretation and meaning-making processes of individual film scenes (Coëgnarts, 2019, 2020; Coëgnarts, 2023a; Coëgnarts, 2023b; Coëgnarts and Kravanja, 2012, 2015; Coëgnarts and Slugan, 2022).

### 4.1 Case-study 1: *Mouchette* (Bresson, 1967)

**Figure 5:**
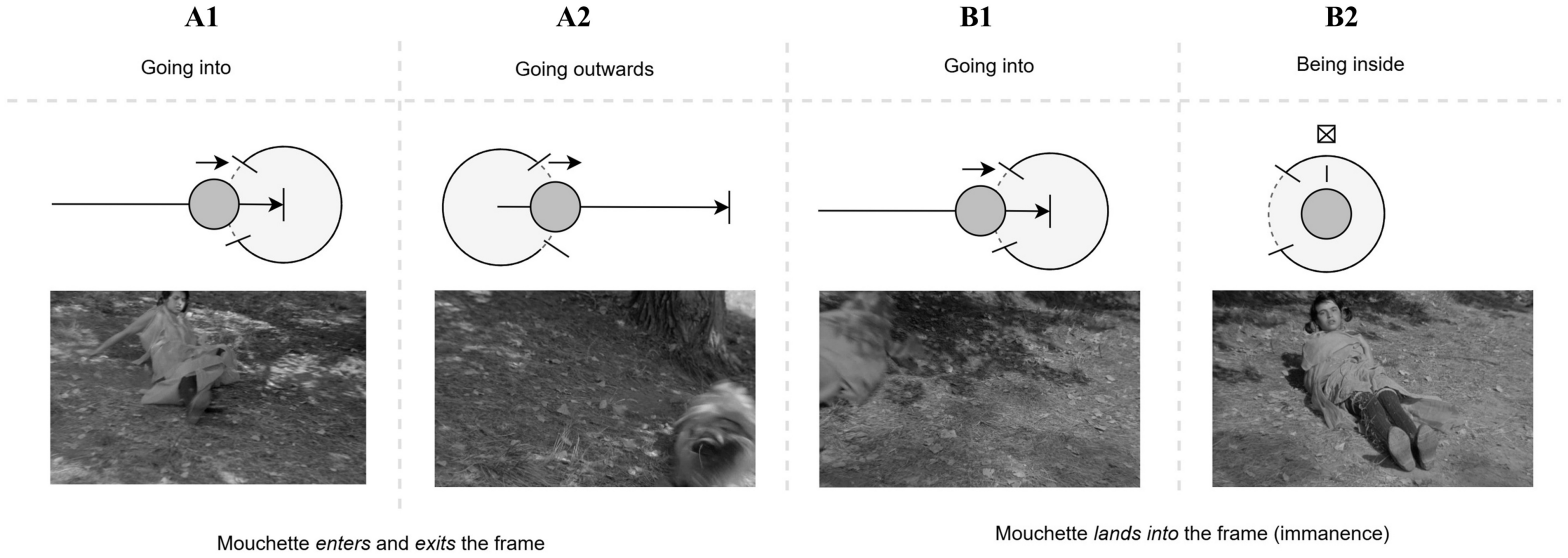
Sequence of shots from *Mouchette* (1967) illustrating the first suicide attempt, where Mouchette rolls into and out of a single shot, coming to rest within the visual content of the frame (still B2). The predictable entry and exit dynamics reflect the container logic of the scene, reinforcing the viewer’s expectation that her trajectory will conclude within the frame.

**Figure 6:**
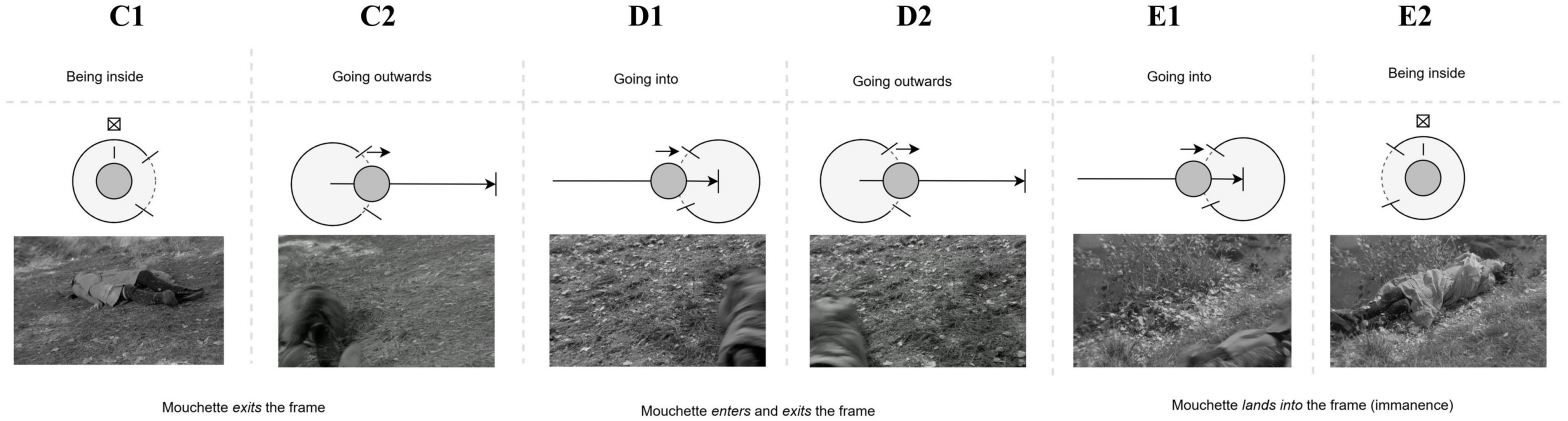
Sequence of shots from *Mouchette* (1967) depicting the second suicide attempt, similar to the first. Mouchette again rolls into and out of the frame, coming to rest within its confines (still E2). This repeated pattern of entry and exit maintains a sense of predictability, mirroring the viewer’s anticipation of her movement within the visual space, further emphasizing the container logic driving the sequence.

Robert Bresson’s *Mouchette* (1967) tells the story of a 14-year-old girl who endures a cold, indifferent world. After a series of tragic events, including the death of her mother, she chooses to end her life (“leave the material world”) by inexplicably committing suicide by rolling down a hill into a pond. This act, the film’s last scene (as also discussed in Coëgnarts, 2022, p.180-181), is depicted through a sequence of shots that each depict a suicide attempt, with the third ultimately succeeding, culminating in a final moment of cinematic “transcendence” (Schrader, 2018). The first two shot sequences, shown in Figures 5, 6, are presented in a similar manner: in both cases, Mouchette rolls *into* and *out of* single shots, each time coming to rest within the visual content of the frame (still **B2** and still **E2**, respectively). The underlying container logic consists of a combination of two dynamic patterns: entry (“going into”) and exit (“going outwards”), which can be represented through the formal language of DISL, and which creates a high sense of predictability. As viewers, we anticipate her movement (or trajectory) concluding within the frame.

Both series of shots align with our expectations of continuity and spatial organization in the context of continuity editing, in particular the cut-on-action technique. In this context, where a cut occurs during a character’s action (entry or exit), the brain is anticipating the completion of the action from one shot to the next. The cut occurs at a point when the viewer’s expectations about the ongoing action are still active, and this helps maintain perceptual continuity, despite the sudden change in the image or perspective (Smith, 2012). From a cognitive perspective, the brain predicts the trajectory of the action, so when the cut happens mid-motion, it is more likely to “fill in the gaps,” making the transition feel seamless.

**Figure 7:**
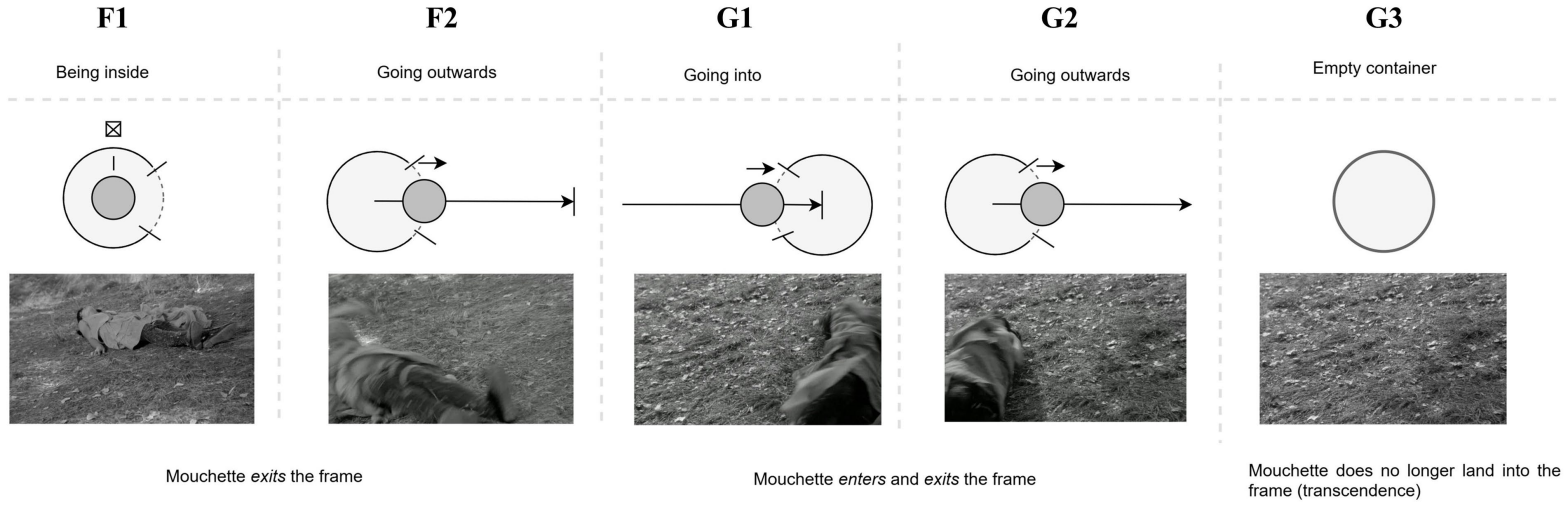
Sequence from *Mouchette* (1967) depicting the third suicide attempt, where the typical entry and exit pattern is disrupted. After Mouchette’s exit from shot (G2), the frame momentarily lingers empty (G3), creating a prediction error. The absence of her physical presence is filled by the acousmatic sound of her fall, which engages the viewer’s interpretative process.

However, as the third attempt unfolds, our predictive model is disrupted as the girl’s exit from the second shot (shot **G2**) this time does not immediately predict the entrance of the third, but instead, the film momentarily lingers on the empty frame (**G3**), allowing the viewer to experience a prediction error. This disruption, arising from the comparison between the perceptual input of the third attempt and that of the preceding ones, draws the viewer-listener into a deeper interpretative engagement with the scene’s content. As Bordwell (2010, p. 5) suggests, viewers rely on their ability to “frame expectations and to be surprised when those are fractured,” a cognitive process vividly illustrated by the lingering empty frame and the acousmatic shift in sound (Chion, 2019). The first two attempts were unsuccessful because Mouchette remained visible to the viewer: the source-path-goal schema guiding her movement concludes twice within the confines of the frame. As such she has not yet transcended the immanent world. By contrast, at the third attempt, the frame is empty and the sound is no longer visually motivated, thus embodying Bresson’s (1975, p. 34) own assertion: “[w]hen a sound can replace an image, cut the image or neutralize it.” The auditory cue—the sound of water splashing—fills the gap left by the missing image, a process central to the PP framework. Sound invites the listener-viewer to “construe” or “complete” what was shown before, the last concluding moment of her physical path, which here finally overlaps with her physical departure. When the films cuts to its final shot we witness the aftermath of her fall into the water. As the river’s surface slowly calms, mirroring the earlier peaceful image seen through the girl’s eyes, the literal sound of the scene’s diegetic world gives way to non-diegetic music—Claudio Monteverdi’s *Magnificat*—which further heightens the transcendental experience. The film, by manipulating both visual and auditory cues, thus activates the viewer’s faculties to “judge,” as Bordwell (2010, p. 5) writes, “what’s presented and then rethink that judgment,” leaving them in a contemplative space as the diegetic world dissolves into the transcendental abstraction of Monteverdi’s *Magnificat* (Figure 7).

### 4.2 Case-study 2: *La Notte* (Antonioni, 1961)

**Figure 8:**
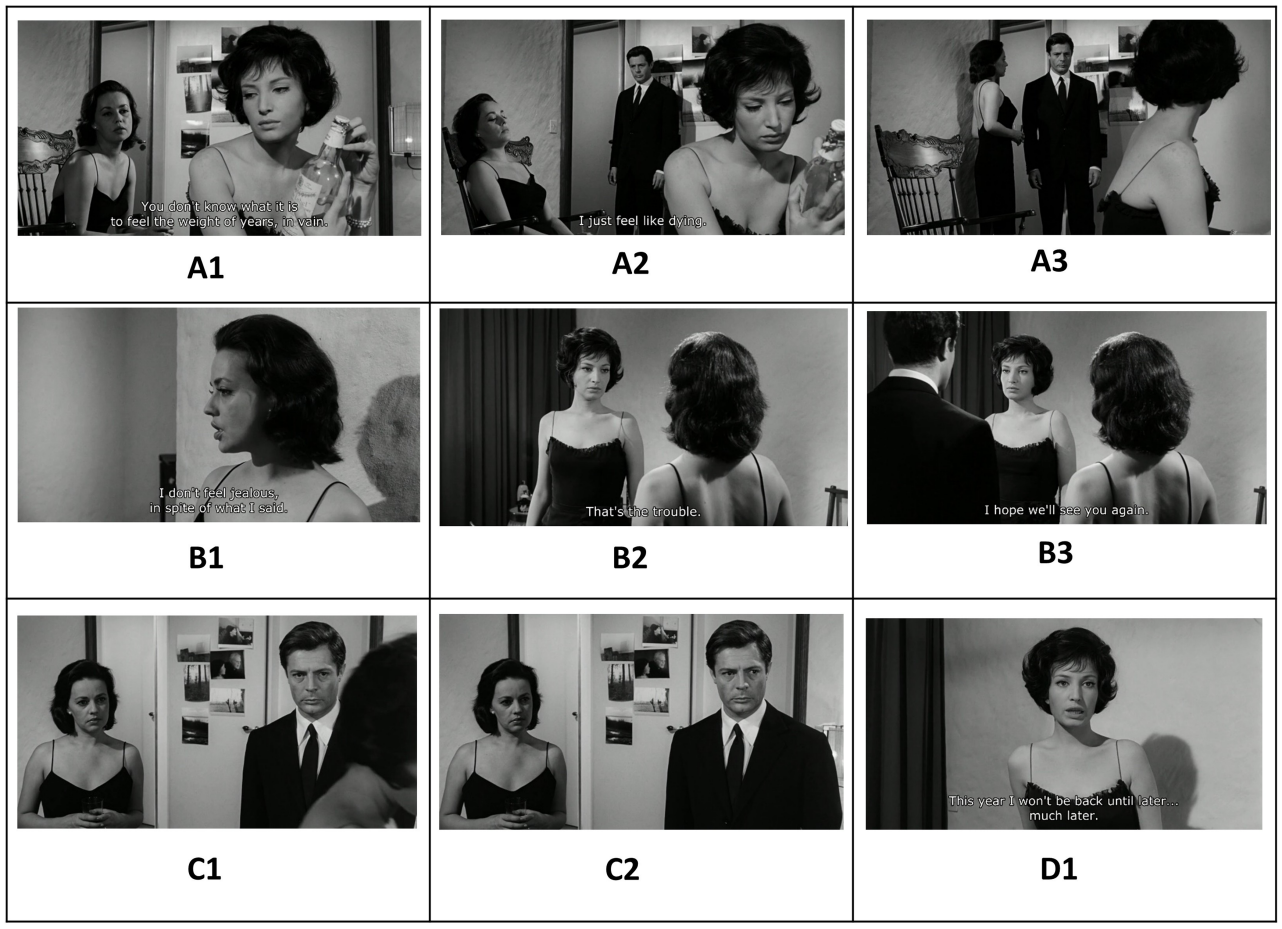
Sequential screenshots from *La Notte* (1961) depicting the evolving spatial dynamics between Lidia, Valentina, and Giovanni. The initial wide framing **(A1)** creates an anticipatory void, soon filled by Giovanni’s entrance from behind the set, forming a visually cohesive triangular structure **(A2)**. Lidia moves towards her husband in the back **(A3)**. The film then cuts to Lidia in medium shot **(B1)**. As the composition shifts, Lidia’s *including* movement toward Valentina **(B2)** and Giovanni’s subsequent entry **(B3)** establish a new triangular configuration, reinforcing a container dynamic in which Valentina is enclosed between the couple. This visual entrapment builds tension, ultimately leading to Valentina’s exit **(C1)**, which dissolves the triangle and restores the couple’s isolated framing **(C2)**. The final shot **(D1)** further emphasizes spatial separation, mirroring the emotional distance between the characters.

**Figure 9:**
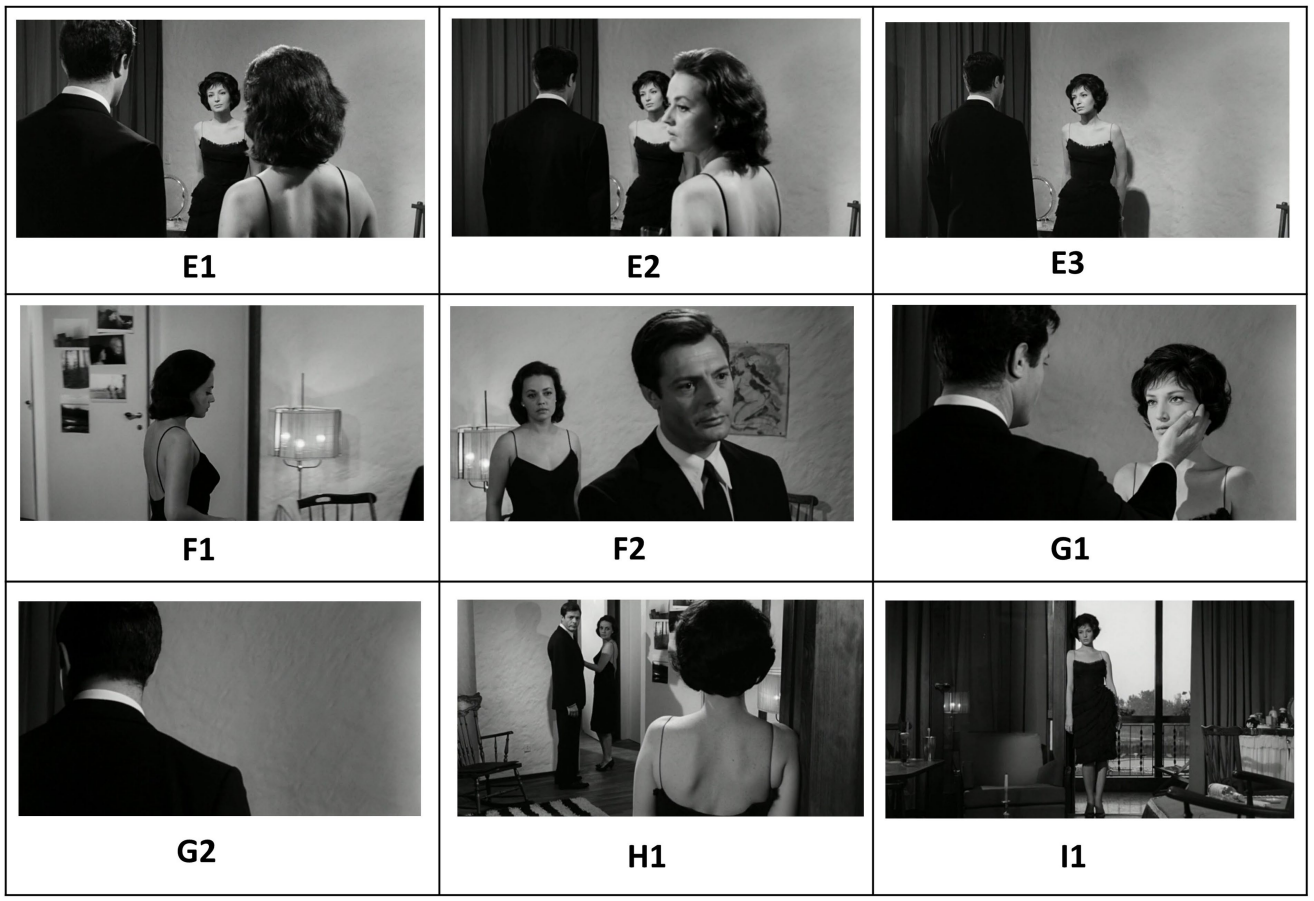
The reestablished triangular composition **(E1)** introduces a greater foreground-background separation, foreshadowing Giovanni’s movement toward Valentina **(E2)**. Lidia’s subsequent exit **(E3)** temporarily isolates the two, but her swift return **(F1)** reclaims Giovanni within her symbolic container **(F2)**. The film then changes perspective by cutting to a two shot of Giovanni and Valentina **(G1)**. As the tension escalates, Valentina, under the weight of Lidia’s presence, withdraws again **(G2)**. In the final frame **(I1)**, she stands alone, delivering her resigned remark (“You’ve exhausted me, the pair of you.”) —an emotional and spatial resolution that encapsulates the film’s themes of exhaustion and detachment.

*La Notte* (1961) is the second film in Michelangelo Antonioni’s “trilogy of alienation,” preceded by *L’Avventura* (1960) and followed by *L’Eclisse* (1962). Set over the course of a single day and night, the film examines the relationship between Giovanni (Marcello Mastroianni), a successful novelist, and his wife, Lidia (Jeanne Moreau), as they confront their growing emotional distance. The narrative takes a turn with the introduction of Valentina (Monica Vitti), the enigmatic daughter of an industrialist whose appealing presence adds complexity to the couple’s relationship. In a key scene, here illustrated in Figures 8, 9, the three characters find themselves sharing the same room as a tense and dynamic interplay begins to unfold. Similar to *Mouchette*, the scene’s thematic content is motivated by an underlying dynamic container logic that initiates a hermeneutic circle of sense-making. Let us have a closer look.

The scene starts off with a conversation between Lidia and Valentina. In one shot, both characters are framed together: Lidia occupies the middle ground on the left, while Valentina is positioned in the foreground on the right (**A1**). However, the widescreen aspect ratio (1.85:1) creates a sense of emptiness in the background of the wide cinematic space. As viewers, we intuitively anticipate that this void will soon be filled. This expectation is met in the following shot when Giovanni *enters* the room from the center background, completing the composition and forming a triangular bounded structure in the formal design (**A2**). We are invited to organize the fragmented sensory information into a Gestalt, a coherent and meaningful whole, a phenomenon known in perceptual psychology as psychological closure (Zettl, 2017, p. 123). The triangular composition is disrupted when the film cuts to a new shot (**B1**). Lidia moves toward Valentina, visually *including* her within her “container” (**B2**). Once again, the widescreen aspect ratio suggests the potential to fill the empty space on the left. This expectation is fulfilled as Giovanni *enters* the frame from the left, creating a new triangular configuration (**B3**). This time, Valentina occupies the center of the frame, with the gaze lines of the couple directing attention toward her. A container dynamic emerges, as Valentina becomes symbolically *enclosed* by the couple (**B3**). This compositional arrangement creates a sense of entrapment that prompts Valentina’s eventual *exit* from the frame (**C1**), which breaks the triangular form and leaves the couple again alone in “their” frame (**C2**). The next shot cuts again to Valentina which further consolidates the spatial separation (**D1**).

This separate framing, though, is short lived as the film revisits the over-the-shoulder shot, reestablishing the triangular composition (**E1**). However, this time, the distance between the foreground (where Giovanni and Lidia are located) and the background (where Valentina is positioned) is noticeably greater. This visual spacing anticipates the movement along the z-axis in the next shot, as Giovanni moves closer in depth to Valentina (**E2**). This action prompts Lidia to *exit* the frame on the right, leaving Giovanni and Valentina alone within the shot (**E3**). However, the film quickly cuts back to Lidia (**F1**), showing the camera following her movement as she reclaims (includes) her husband again *into* her personal “container,” thereby reasserting her dominance within the love triangle (**F2**). The film then cuts to a medium shot of Giovanni and Valentina (**G1**). Feeling the “pressure” of Lidia, she now *exits* the frame again (**G2**). At the end she is standing alone (**H1**), while uttering the words: “You’ve exhausted me, the pair of you.”

**Figure 10:**
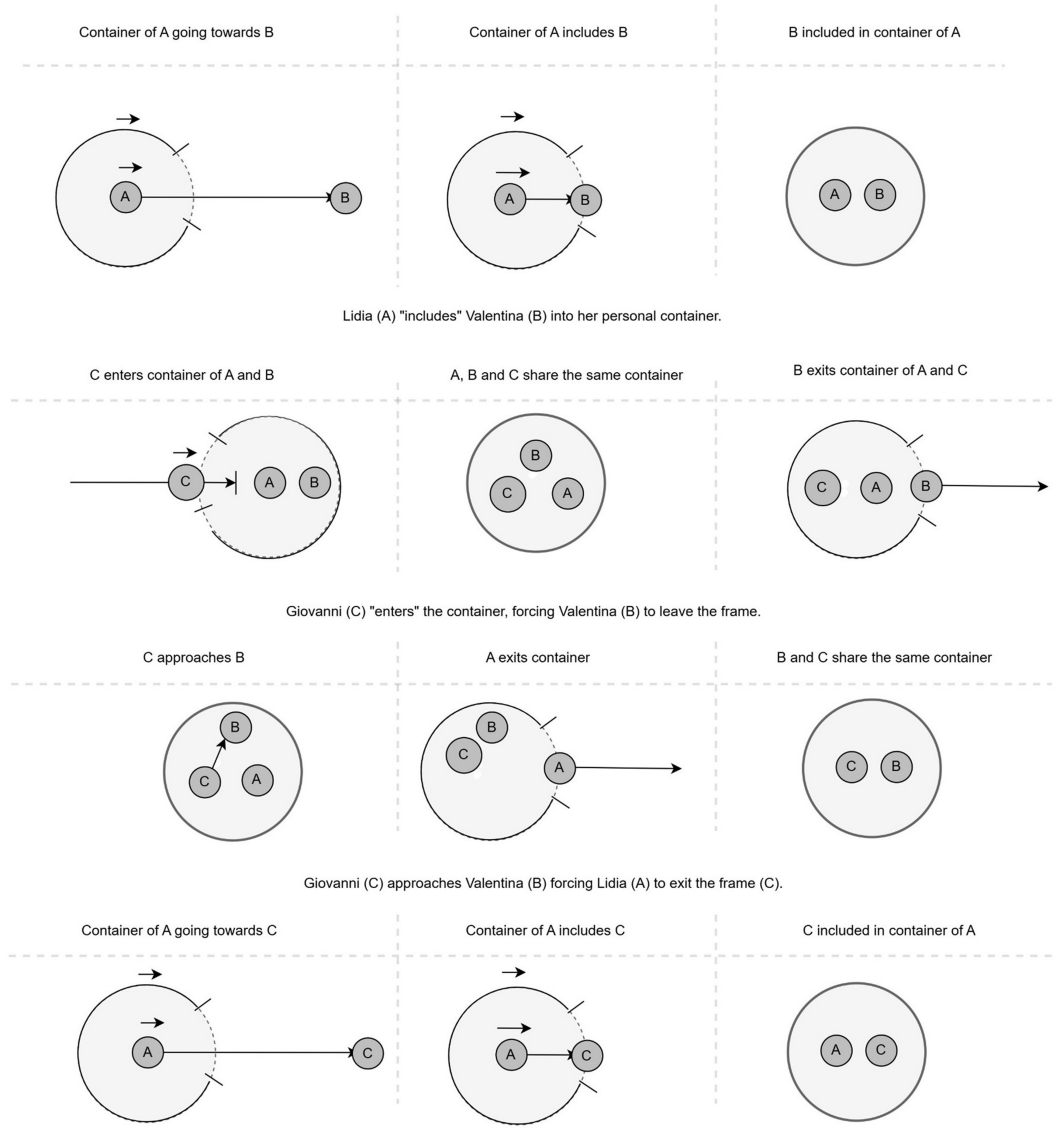
Diagrammatic representation of the dynamic container logic underlying the scene in *La Notte*. This visual summary maps the evolving spatial configurations, movement patterns, and shifting container relationships between the characters.

As was the case in *Mouchette*, predictive processing shapes the viewer’s engagement by guiding expectations through spatial composition and character movement. The dynamic interplay of framing and re-framing—particularly the evolving triangular configurations—creates a structured yet fluid perceptual experience, compelling the viewer to anticipate changes in spatial relationships. Each disruption, whether through character entrances, exits, or shifts in depth, generates a prediction error that invites deeper cognitive and emotional engagement with the scene’s underlying tensions. As Valentina ultimately withdraws, her final statement underscores the culmination of this interplay: a moment of exhaustion and disillusionment that reflects the film’s broader meditation on alienation and emotional disconnect. Dynamic patterns of containment play a significant role in structuring this higher-order semantic content, as spatial relationships between characters evoke deeper metaphorical meanings (Coëgnarts, 2019, 2020). The container logic governs the shifting dynamics of inclusion and exclusion, with Valentina repeatedly positioned within and then expelled from the couple’s symbolic space. Similarly, the path schema underlies the directional movements of the characters, shaping the viewer’s expectations about who will enter, exit, or shift within the frame. These schema-driven perceptual cues, when momentarily disrupted, create cognitive dissonance that mirrors the emotional instability of the characters themselves. Diagrammatically, we may represent and visually summarize the entire dynamic logic underlying the scene as described above as in Figure 10.

## 5 Concluding remarks and future directions

This article highlights the potential of integrating predictive processing and image schema theory to deepen our understanding of meaning-making in cinematic art works. By framing films as deliberate activations of the brain’s inferential processes, we demonstrated how cinematic works align sensory input with the predictive logic of the mind, enhancing comprehension and higher-level semantic engagement. In this sense our study adheres to the broader conclusion made by Frascaroli (2022, p. 134), namely “that effective artworks are crafted to offer beings like us a rich supply of graspable causal structures, making them experience a consistent feeling of cognitive gain.” Image schemas, as dynamic patterns linking sensory-motor interactions to abstract reasoning, provide a valuable lens for analyzing, in a more fine-grained manner, how films engage viewers’ inferential embodied cognitive architecture. As such, this article establishes predictive processing as a fruitful paradigm for advancing inferential models in cognitive film theory. Only fairly recently, it was claimed that “film and media cognitivism now rarely refers to inferential (let alone computational) models of mental activity” (Nannicelli and Taberham, 2014, p. 5). Instead, greater emphasis has been placed on affect-driven mental processes (Grodal, 1999; Plantinga and Smith, 1999; Smith, 1995; Tan, 1996). The PP framework, however, has the potential to bridge these two strands, integrating both “cold cognition” and “hot cognition” into a unified framework.

Building on these findings, future research can extend this framework in several promising directions. First, recasting classical cognitive film theory through the lens of predictive processing offers a fresh perspective on the viewer’s active role in constructing cinematic meaning. Just as Seth and Kenser aligned Gombrich’s theories with the prediction error minimization framework, key theoretical accounts by scholars such as David Bordwell and Julian Hochberg—focused on inferential processes and mental representation in narrative comprehension and film perception—could similarly benefit from an analysis of how predictive mechanisms enhance cognitive, meaning-making processing during film viewing. Second, situating cinema within the recent debate on aesthetic cognitivism provides an opportunity to explore how films contribute to cognitive enrichment. Aesthetic cognitivism posits that art, and in particular fiction, has cognitive value, not just as a source of pleasure but as a means of imparting (non-trivial) knowledge and fostering understanding (Baumberger, 2013; Christensen et al., 2023; Gaut, 2005; Graham, 1996; Schellekens, 2024; Vidmar Jovanović, 2023). Hence, by engaging viewers’ inferential capacities, films not only evoke emotional and aesthetic responses but also facilitate deeper understanding and intellectual engagement, positioning cinema as a cognitively enriching art form. This also aligns well with current accounts of aesthetic education, such as Nannicelli’s (2024) perceptual-cognitive model, which reframes aesthetic education as a process centered on developing perceptual and cognitive capacities rather than merely refining taste. Finally, the rich line of research on complex “puzzle films” and “loop narratives” which challenge audiences with fragmented narratives and ambiguous resolutions, highlights the critical role of prediction in film aesthetics (Kiss and Willemsen, 2017; Willemsen, 2025; Willemsen and Kiss, 2022). These films demand that viewers anticipate, revise, and resolve narrative trajectories, offering a fertile testbed for investigating predictive inference in dynamic contexts.

A crucial question for future research is determining where within this hierarchy image schemas function in relation to predictive processing. Do cinematic formal structures primarily engage unconscious sensorimotor patterns, or do they operate at a higher level, facilitating conceptual mechanisms for narrative comprehension and emotional engagement? Additionally, for a PP model based on image schemas to be empirically viable, a clear methodological framework is needed to measure predictive processes across different levels of abstraction.

Another key avenue for future research lies in exploring the role of multisensory integration in cinematic prediction. Film is inherently multimodal, incorporating visual, auditory, and linguistic elements (Bateman and Schmidt, 2013; Tseng et al., 2021). Empirical research on prediction mechanisms across different sensory modalities could help clarify how viewers integrate information from different perceptual streams to generate coherent expectations about cinematic narratives. By incorporating findings from cross-modal predictive processing studies, future work can further illuminate the relationship between sensory input and inferential meaning-making, demonstrating how predictive models extend beyond visual narrative comprehension to a fully multimodal cinematic experience.

Moreover, advancing this research requires methodologies that can effectively capture and communicate the dynamic nature of image-schematic patterns in cinematic sequences. In this regard, graphic animations serve as crucial tools for visualizing these evolving structures, offering a creative means to represent complex spatial and temporal relationships that shape predictive engagement. By rendering movement, containment, and force dynamics in a schematic yet perceptually salient manner, such visualizations align with the need for alternative annotation systems in film analysis—an idea already suggested by Hochberg and Brooks (2007, p. 390): “we need an annotation system better fitted by visual displays than by words. Perhaps it should consist of brief high points or action features economically sampled from the flow of events; it will be relatively schematic, since details are not normally maintained unless needed; it will be mostly ego-centered or camera-centered, with a definite viewpoint and 2D composition.” This perspective also resonates with ongoing developments in artistic research and video-graphic film criticism (e.g., Coëgnarts, 2023b; Kiss and Coëgnarts, 2025; Scott, 2024), where visualization techniques are employed not merely as explanatory tools but as creative and epistemic instruments in their own right. Future studies that integrate predictive processing, cognitive film theory, and artistic research methodologies could further elucidate the ways in which cinematic form interacts with human cognition, refining both theoretical models and artistic practice.

## Data Availability

The original contributions presented in the study are included in the article/supplementary material, further inquiries can be directed to the corresponding author/s.
